# Analysis of risk factors for severe acute kidney injury in patients with acute myocardial infarction: A retrospective study

**DOI:** 10.3389/fneph.2023.1047249

**Published:** 2023-02-09

**Authors:** Yuxin Nong, Xuebiao Wei, Hongrui Qiu, Honghao Yang, Jiale Yang, Junquan Lu, Jianfeng Cao, Yanbin Fu, Danqing Yu

**Affiliations:** ^1^ Guangdong Cardiovascular Institute, Guangdong Provincial People's Hospital, Guangdong Academy of Medical Sciences, Guangzhou, China; ^2^ Guangdong Provincial Key Laboratory of Coronary Heart Disease Prevention, Guangdong Provincial People's Hospital, Guangdong Academy of Medical Sciences, Guangzhou, China; ^3^ Department of Geriatric Intensive Medicine, Guangdong Provincial Geriatrics Institute, Guangdong Provincial People's Hospital, Guangdong Academy of Medical Sciences, Southern Medical University, Guangzhou, China; ^4^ Department of Thoracic Surgery, Guangdong Provincial People's Hospital, Guangdong Academy of Medical Sciences, Guangzhou, China; ^5^ Zhongshan School of Medicine, Sun Yat-sen University, Guangzhou, China

**Keywords:** myocardial infarction, acute kidney injury (AKI), risk factors, mortality, prognosis

## Abstract

**Background:**

Patients with acute myocardial infarction (AMI) complicated by acute kidney injury (AKI) tend to have a poor prognosis. However, the exact mechanism of the co‐occurrence of the two diseases is unknown. Therefore, this study aims to determine the risk factors for severe AKI in patients with AMI.

**Methods:**

A total of 2022 patients were included in the Medical Information Mart for Intensive Care. Variables were identified *via* univariate logistic regression, and the variables were corrected *via* multivariate logistic regression. Restricted cubic splines were used to examine the risks associated with the variables. The Kaplan–Meier method was used to compare the risk of severe AKI among the patients.

**Results:**

Patients with severe AKI had a higher in‐hospital mortality rate (28.6% vs. 9.0%, P < 0.001) and a longer duration of intensive care (6.5 days vs. 2.9 days, P < 0.001). In patients with AMI, the mean systolic blood pressure (SBP); international normalized ratio (INR); the levels of blood urea nitrogen (BUN), glucose, and calcium; and a history of liver disease were found to be the independent risk factors for developing severe AKI after their admission. Increased levels of BUN and blood glucose and a high INR increased the risk of severe AKI; however, increased levels of calcium decreased the risk; SBP presented a U‐shaped curve relationship.

**Conclusions:**

Patients with severe AKI have a poor prognosis following an episode of AMI. Furthermore, in patients with AMI, SBP; INR; a history of liver disease; and the levels of BUN, glucose, and calcium are the independent risk factors for developing severe AKI after their admission.

## 1 Introduction

Acute myocardial infarction (AMI) is one of the most common cardiovascular diseases in the world. During the past decade, population growth and aging have increased the incidences of ischemic cardiomyopathy worldwide, reaching 182 million in 2019 (1). A large sample multi‐center retrospective study based on a national database has shown that organ failure as a result of hypoperfusion after AMI increases the risk of cardiac arrest and in‐hospital mortality in patients with AMI as well as hospitalization costs and duration of hospital stay (2).

In patients with AMI, acute kidney injury (AKI) is a common complication (3). AKI in the early stages is associated with poor outcomes. Among patients with AMI complicated by AKI, two‐thirds develop AKI within the initial days of admission (4).

However, there is no recognized early warning mechanism for AKI after an episode of AMI. The pathophysiology of AKI is affected by many factors; patients have different prognostic risks at different stages. Patients with transient or mild AKI recover quickly after treatment, and their long‐term mortality risk is similar to that of patients with no AKI. Nevertheless, patients with severe AKI are at an increased risk of short‐term adverse events, such as acute pulmonary edema, ventricular arrhythmia, and high in‐hospital mortality (5). Therefore, early identification and timely treatment of severe AKI are crucial for patients with AMI complicated by AKI (6).

In this study, we retrospective analysis of clinical factors that could predict acute kidney injury following acute myocardial infarction.

## 2 Materials and methods

### 2.1 Study cohort

The clinical data were collected from Medical Information Mart for Intensive Care IV (MIMIC IV clinical database), a large real‐world medical database, to record the information of patients admitted to critical care units at the Beth Israel Deaconess Medical Center between 2008 and 2019. The database includes medical data, such as demographics, vital sign measurements, laboratory test results, and medical procedures, of patients during hospitalization. International Classification of Diseases diagnosis 9 codes was used to identify and extract information about the first hospitalizations of patients diagnosed with AMI. The inclusion criteria included: 1. age ≥18 years and 2. diagnosis was AMI (ICD9: 410.00−410.92, see **Supplementary Table 1**). The exclusion criteria included: 1. kidney function could not be assessed, 2. records of a history of chronic kidney disease (CKD) (ICD9: 585.1‐585.9). The above data were extracted and sorted through the software pgAdmin (6.14).

The study was approved by the Institutional Review Boards of Beth Israel Deaconess Medical Center (Boston, MA) and the Massachusetts Institute of Technology (Cambridge, MA). The study did not impact clinical care and all protected health information was unidentified. Therefore, patient consent was not required. The relevant database descriptions have been described previously (7).

### 2.2 Outcomes

The primary outcome was the diagnosis of AKI in stage 3 in the first 7 days after admission, according to the Global Outcomes Organization for Kidney Disease: Improving Global Outcomes‐AKI (KDIGO‐AKI) (8). In this study, urine volume is subjective to external influences and interventions (such as transfusions); therefore, only blood creatinine level was used as a diagnostic marker for AKI. Additionally, because the diagnostic criteria for AKI in some patients admitted earlier may differ from the current criteria (KDIGO‐AKI guidelines were published in 2012), a new algorithm was used to recalculate creatinine levels and diagnose AKI in patients with AMI. Creatinine baseline was defined as the first measurement, taken 6 h before and within 24 h after admission to the intensive care unit (ICU). AKI is diagnosed when creatinine levels 1.5 times the baseline within 7 days after admission or >0.3 mg/dl within 48 h. Creatinine levels 3 times the baseline or >4.0 mg/dl is defined as stage 3 AKI. All the above diagnostic codes are available on MIMIC’s website following the instructions (https://mimic.mit.edu/). The time from admission to discharge was defined as the survival time when a patient died in the hospital, otherwise, it was considered follow‐up cut‐off time.

### 2.3 Variables

The variables collected from the database included patient demographics: age, gender, admission type, race, and marital status. The mean of results from laboratory tests, hematocrit, hemoglobin, platelet, anion gap, bicarbonate, blood urea nitrogen (BUN), calcium, chloride, sodium, potassium, creatinine, glucose, admission creatinine, white blood cell, international normalized ratio (INR), and oxygen saturation, conducted on the first day of admission was recorded. The mean of vital signs, heart rate, respiratory rate, temperature, systolic blood pressure (SBP), and diastolic blood pressure (DBP), was recorded on the first day of admission. The complications in hospitalized patients included congestive heart failure, cerebrovascular disease, chronic obstructive pulmonary disease (COPD), liver disease, hemiplegia or paraplegia, and cancer. The type of admission, the duration of stay, and the duration in the ICU were recorded. The remaining complications in the currently hospitalized patients were obtained using text codes from discharge diagnosis records; see **Supplementary Table 2**.

### 2.4 Statistical analysis

The variables were presented as mean ± standard deviation and compared *via* the Mann–Whitney U test or student’s t‐test. The categorical variables were presented as numbers and percentages and were tested using the chi‐squared test. Variables missing <20% were filled by multiple interpolations (9), see **Supplementary Table 3**. Kaplan-Meier survival analysis and log‐rank test were used to compare the survival prognosis of the two groups. The variables were first analyzed using the univariate logistic regression analysis. Thereafter, the significant variables were divided into three quantiles and included in multivariate logistic regression analysis. A restricted cubic spline (RCS) curve was used to evaluate the risk curve. All the above statistical analyses were performed *via* the R software (version 4.1, https://www.r‐project.org/). A two‐sided test with a P value < 0.05 was considered statistically significant.

## 3 Results

### 3.1 Patient characteristics

**Figure 1:**
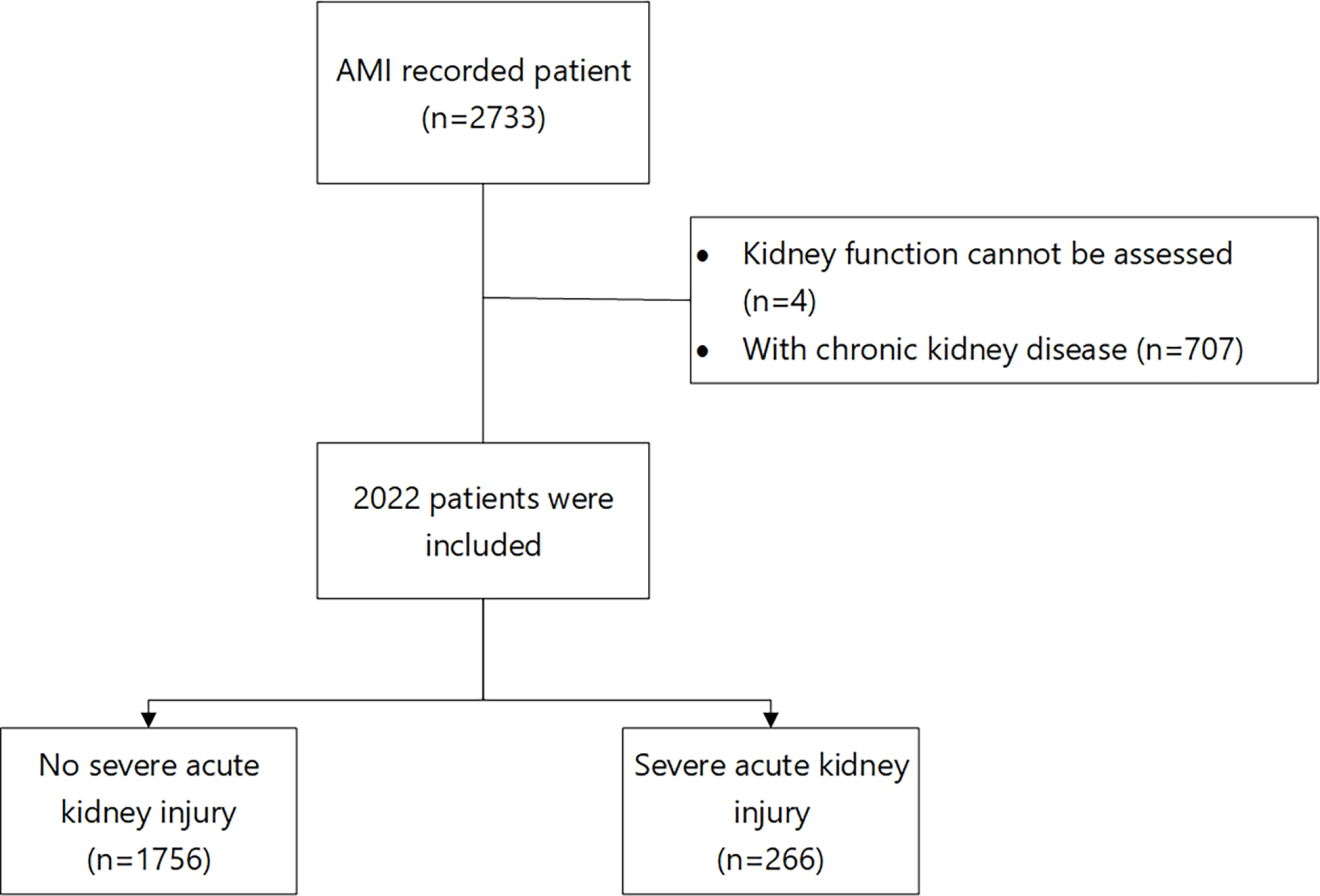
Patient enrollment process.

A total of 3675 patients were obtained from the records. Out of which, 2022 patients, including 1258 (62.2%) men and 764 (37.8%) women, were enrolled in the study after the exclusion; 266 (13.2%) patients had severe AKI. The enrollment process is shown in **Figure 1**. The baseline characteristics of the two groups are shown in **Table 1**.

**Table 1:** Clinical characteristics of patients.

		severe acute kidney injury		
	Overall	No	Yes	P valve
	n=2022	n=1756	n=266	
Age (year)	68.97 (13.37)	68.99 (13.29)	68.83 (13.86)	0.864
Gender (%)				
Women	764 (37.8)	657 (37.4)	107 (40.2)	0.416
Men	1258 (62.2)	1099 (62.6)	159 (59.8)	
Race (%)				
Asian	42 (2.1)	37 (2.1)	5 (1.9)	0.695
Black	93 (4.6)	77 (4.4)	16 (6.0)	
Unknown	465 (23.0)	405 (23.1)	60 (22.6)	
White	1422 (70.3)	1237 (70.4)	185 (69.5)	
Marital status (%)				
Divorced	134 (6.6)	115 (6.5)	19 (7.1)	0.042
Married	1051 (52.0)	934 (53.2)	117 (44.0)	
Unknown	166 (8.2)	136 (7.7)	30 (11.3)	
Single	385 (19.0)	324 (18.5)	61 (22.9)	
Widowed	286 (14.1)	247 (14.1)	39 (14.7)	
Admission type (%)				
Elective	16 (0.8)	14 (0.8)	2 (0.8)	0.660
Emergency	1075 (53.2)	924 (52.6)	151 (56.8)	
Surgical	41 (2.0)	36 (2.1)	5 (1.9)	
Urgent	890 (44.0)	782 (44.5)	108 (40.6)	
Hematocrit (%)	34.45 (5.79)	34.63 (5.76)	33.30 (5.92)	<0.001
Hemoglobin (g/dL)	11.61 (2.04)	11.70 (2.03)	11.05 (2.00)	<0.001
Anion gap (mmol/L)	14.20 (3.43)	13.88 (3.22)	16.36 (3.95)	<0.001
Bicarbonate (mmol/L)	23.51 (3.71)	23.88 (3.45)	21.06 (4.39)	<0.001
Blood Urea Nitrogen (mg/dl)	22.26 (14.54)	20.67 (12.05)	32.72 (22.89)	<0.001
Calcium (mg/dl)	8.46 (0.73)	8.52 (0.70)	8.05 (0.82)	<0.001
Chloride (mmol/L)	104.69 (4.67)	104.60 (4.45)	105.28 (5.92)	0.026
Creatinine (mg/dl)	1.10 (0.96)	1.01 (0.48)	1.68 (2.25)	<0.001
Glucose (mg/dL)	150.17 (62.07)	144.16 (55.23)	189.86 (85.80)	<0.001
Sodium (mmol/L)	138.06 (3.73)	138.03 (3.56)	138.24 (4.66)	0.395
Potassium (mmol/L)	4.20 (0.47)	4.17 (0.45)	4.33 (0.59)	<0.001
International normalized ratio (s)	1.37 (0.61)	1.34 (0.56)	1.59 (0.81)	<0.001
Heart rate (bmp)	81.82 (14.37)	81.15 (13.84)	86.22 (16.89)	<0.001
Systolic blood pressure (mmHg)	114.50 (13.85)	114.77 (13.63)	112.75 (15.18)	0.027
Diastolic blood pressure (mmHg)	61.91 (10.62)	62.01 (10.52)	61.21 (11.22)	0.250
Respirate rate (bmp)	19.11 (3.39)	18.90 (3.18)	20.54 (4.29)	<0.001
Temperature (°C)	36.73 (0.63)	36.73 (0.56)	36.67 (0.97)	0.101
SPO2 (mmHg)	96.89 (2.57)	96.89 (2.51)	96.93 (2.94)	0.805
Congestive heart failure (%)				
No	1941 (96.0)	1689 (96.2)	252 (94.7)	0.340
Yes	81 (4.0)	67 (3.8)	14 (5.3)	
Cerebrovascular disease (%)				
No	1823 (90.2)	1598 (91.0)	225 (84.6)	0.002
Yes	199 (9.8)	158 (9.0)	41 (15.4)	
Chronic obstructive pulmonary disease (%)				
No	1510 (74.7)	1327 (75.6)	183 (68.8)	0.022
Yes	512 (25.3)	429 (24.4)	83 (31.2)	
Liver disease (%)				
No	1898 (93.9)	1690 (96.2)	208 (78.2)	<0.001
Yes	124 (6.1)	66 (3.8)	58 (21.8)	
Hemiplegia/paraplegia (%)				
No	2015 (99.7)	1751 (99.7)	264 (99.2)	0.517
Yes	7 (0.3)	5 (0.3)	2 (0.8)	
Cancer (%)				
No	1889 (93.4)	1651 (94.0)	238 (89.5)	0.008
Yes	133 (6.6)	105 (6.0)	28 (10.5)	

SPO_2_, pulse oximeter oxygen saturation; INR, international normalized ratio.

### 3.2 Prognosis of patients with AMI and severe AKI

**Figure 2:**
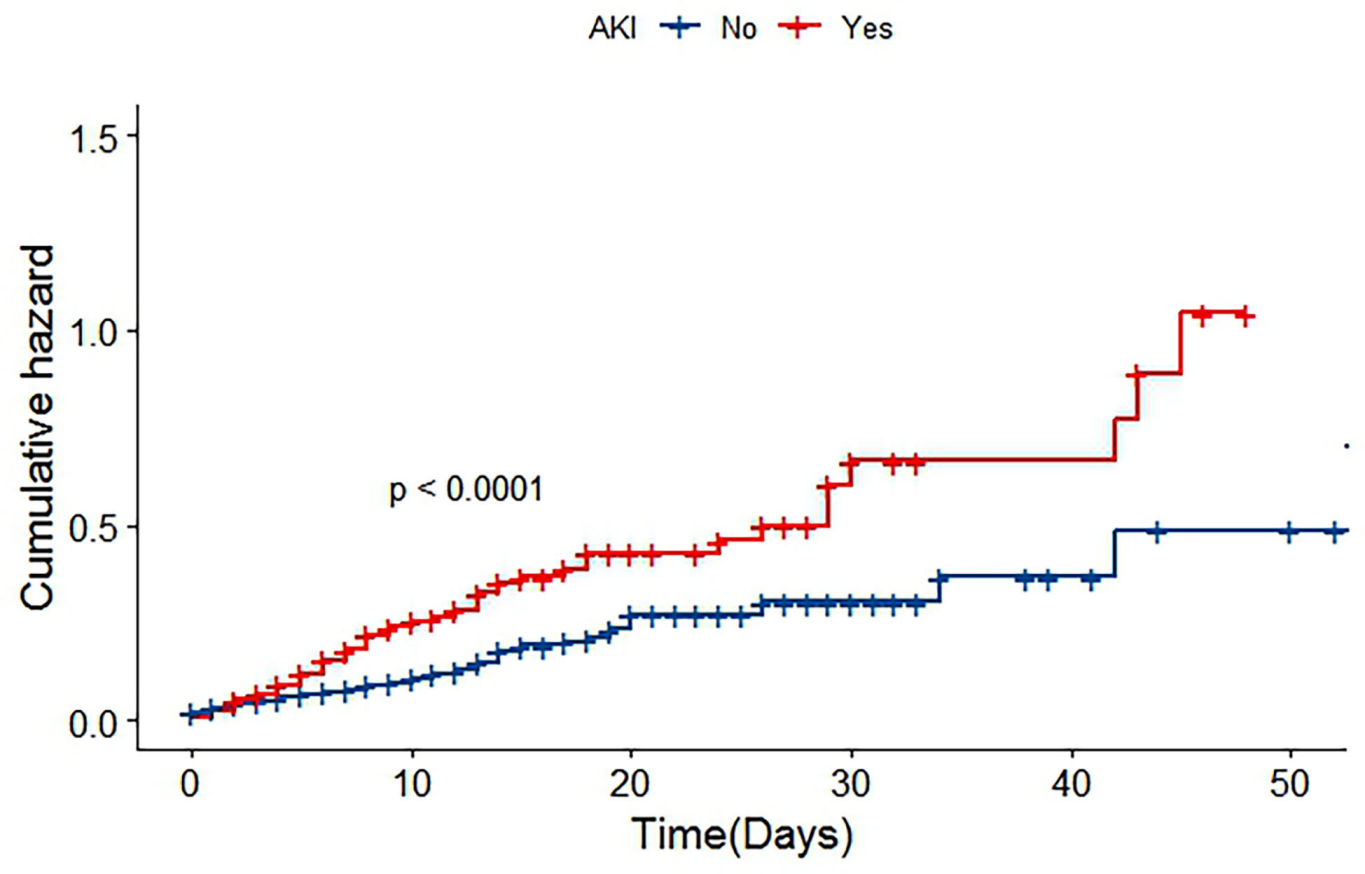
Cumulative risk curve of patients with in-hospital death.

Patients with severe AKI had significantly higher in‐hospital mortality than patients with non‐severe AKI (28.6% vs 9.0%, P < 0.001). The risk of death in the two groups is shown in **Figure 2** (P< 0.001). Additionally, patients with severe AKI spent more time in the ICU than patients without severe AKI (severe AKI vs non‐severe AKI: 6.5 days vs. 2.9 days, P < 0.001).

### 3.3 Risk factors associated with severe AKI

After the univariate logistic regression analysis, the mean of first‐day lab test results of hematocrit, hemoglobin, anions gap, bicarbonate, BUN, calcium, chloride, glucose, potassium, INR, heart rate, SBP and respiration rate and the history of cerebrovascular disease, COPD, liver disease, and cancer were associated with severe AKI, as shown in **Table 2**. SBP, BUN, glucose, INR, and calcium as well as the history of liver disease were the statistically significant independent risk factors after adjusting with multivariate logistic regression, as shown in **Table 3**.

**Table 2:** Univariate logistic regression analysis.

Variables	Unadjusted	95%CI		P valve
	OR	Lower Upper		
Age	0.999	0.989	1.008	0.798
Gender (Men)	0.888	0.683	1.156	0.378
Hematocrit (%)	0.960	0.938	0.982	<0.001
Hemoglobin (g/dL)	1.176	1.101	1.257	<0.001
Anion gap (mmol/L)	1.197	1.154	1.240	<0.001
Bicarbonate (mmol/L)	0.816	0.787	0.846	<0.001
Blood Urea Nitrogen (mg/dl)	1.044	1.035	1.052	<0.001
Calcium (mg/dl)	0.415	0.343	0.501	<0.001
Chloride (mmol/L)	1.032	1.004	1.061	0.027
Glucose (mg/dL)	1.009	1.007	1.011	<0.001
Potassium (mmol/L)	1.915	1.485	2.469	<0.001
International normalized ratio (s)	1.568	1.334	1.843	<0.001
Heart rate (bmp)	1.023	1.014	1.032	<0.001
Systolic blood pressure (mmHg)	0.989	0.980	0.999	0.026
Diastolic blood pressure (mmHg)	0.992	0.980	1.005	0.226
Respirate rate (bmp)	1.132	1.093	1.172	P<0.001
Congestive heart failure	1.401	0.776	2.529	0.264
Cerebrovascular disease	1.843	1.272	2.670	<0.001
Chronic obstructive pulmonary disease	1.403	1.059	1.859	0.018
Liver disease	7.140	4.878	10.452	<0.001
Cancer	1.850	1.193	2.868	0.006

**Table 3:** Multivariable logistic regression analysis.

Variables	adjusted OR	95%CI		P valve
		Lower	Upper	
Hematocrit (%)				
T1	Ref			
T2	0.981	0.555	1.736	0.948
T3	1.106	0.486	2.518	0.810
Hemoglobin (g/dL)				
T1	Ref			
T2	0.847	0.475	1.512	0.575
T3	0.619	0.268	1.431	0.262
Anion gap (mmol/L)				
T1	Ref			
T2	1.306	0.825	2.066	0.254
T3	0.709	0.491	1.023	0.166
Bicarbonate (mmol/L)				
T1	Ref			
T2	0.649	0.436	1.966	0.683
T3	0.612	0.379	2.987	0.724
Blood Urea Nitrogen (mg/dl)				
T1	Ref			
T2	2.036	1.287	3.220	0.002
T3	3.692	2.709	5.023	0.000
Chloride (mmol/L)				
T1	Ref			
T2	0.907	0.608	1.353	0.631
T3	1.395	0.936	2.079	0.102
Calcium (mg/dl)				
T1	Ref			
T2	0.703	0.376	0.937	0.031
T3	0.815	0.601	0.942	0.043
Glucose (mg/dL)				
T1	Ref			
T2	3.389	2.217	4.903	0.005
T3	4.901	3.987	5.107	<0.001
Potassium (mmol/L)				
T1	Ref			
T2	0.902	0.611	1.332	0.605
T3	1.076	0.750	1.544	0.691
International normalized ratio (s)				
T1	Ref			
T2	2.483	2.020	3.627	0.033
T3	3.361	2.305	3.047	0.005
Heart rate (bmp)				
T1	Ref			
T2	1.034	0.697	1.535	0.868
T3	1.197	0.821	1.746	0.349
Systolic blood pressure (mmHg)				
T1	Ref			
T2	1.018	0.781	1.601	0.542
T3	1.571	1.058	2.243	0.028
Respirate rate (bmp)				
T1	Ref			
T2	0.656	0.440	1.978	0.078
T3	0.845	0.579	1.234	0.383
Chronic obstructive pulmonary disease	1.258	0.907	1.745	0.169
Liver disease	3.139	2.014	4.893	<0.001
Cancer	1.228	0.735	2.050	0.433

T, Tertiles of variable.

### 3.4 Association between risk factors and the trend of severe AKI

**Figure 3:**
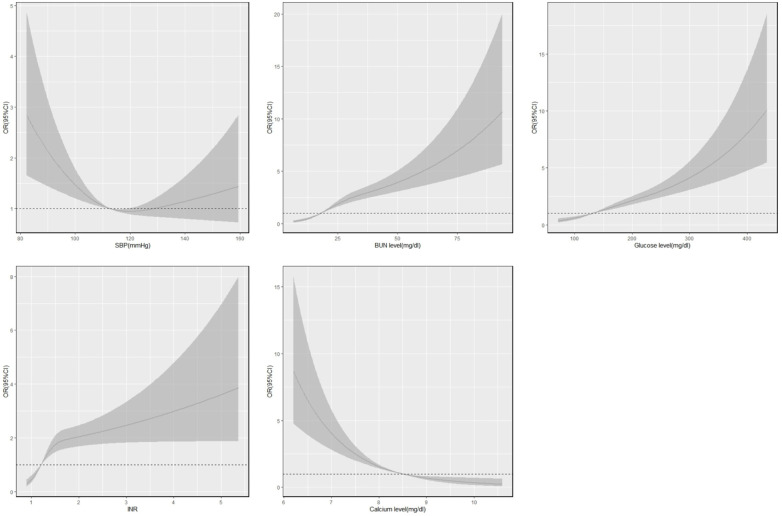
Restricted cubic spline of variables and risk of in-hospital severe acute kidney injury.

An RCS was constructed to analyze the trends in risk factors for severe AKI in patients with AMI in the hospital, as shown in **Figure 3**. The results showed that severe AKI risk increased with an increase in the levels of BUN, glucose, and INR in AMI patients; cut‐off points were 18.4 mg/dl, 131.4 mg/dl, and 1.2 mg/dl, respectively. When INR exceeded the cut‐off point, the risk of severe AKI increased drastically. The risk of severe AKI decreased with increasing calcium levels, with a cut‐off point of 8.5 mg/dl. A U‐shaped relationship was obtained between the risk of severe AKI and SBP, which first decreased with increasing blood pressure and then increased after 112 mmHg but at a relatively slow rate.

## 4 Discussion

Currently, AKI remains one of the most common complications of AMI, and the decline in renal function worsens the prognosis of patients with AMI. The exact mechanism of the co‐occurrence of AKI despite several preventive clinical measures is still unclear (10). In our study, compared with patients without severe AKI, patients with severe AKI had a more than three‐fold higher risk of in‐hospital mortality and approximately two‐fold longer duration of intensive care, which is consistent with previous studies (11). Once AKI progresses to a severe stage, kidney function becomes difficult to recover, and the disease is likely to become irreversible, thereby resulting in considerably poor outcomes and increased medical burden. Therefore, early identification of AKI and prevention of severe AKI are crucial.

In this study, a correlation between high SBP and severe AKI was observed. The risk of severe AKI was found to be associated with SBP, measured during admission, in a U‐shaped pattern. Blood perfusion is one of the leading causes of renal ischemia injury; SBP represents blood flow to some extent. Acute renal ischemia results in the necrosis of tubular epithelial cells and the induction of renal immunoinflammatory responses (12). Therefore, patients with early SBP decline should receive fluid supplementation and be closely monitored for severe AKI. Furthermore, in the present study, the risk for severe AKI increased when blood pressure exceeded 112 mmHg, but the trend was relatively gentle. However, DBP was not a risk factor for severe AKI. Therefore, the findings suggest that AKI is not the main cause of AMI but rather the hemodynamic disorder and the insufficiency of organ blood circulation.

The levels of BUN, a traditional indicator of renal function, has been used as a strong prognostic indicator of the risk of cardiovascular disease (13). Urea is a protein metabolite of creatinine, and BUN levels reflect the balance in its production in the liver and functions of renal excretion (14). In the present study, BUN level was still predictive of severe AKI in patients with AMI after the patients with CKD were excluded and adjusted for liver disease. Moreover, urea itself produces reactive oxygen species and promotes oxidative stress, thereby damaging myocardial and renal tubular epithelium (15, 16).

Hyperglycemia during admission increases the risk of acute kidney damage in patients with AMI (17), which is consistent with the findings of the present study. The incidence of adverse events during hospitalization is higher after an episode of MI as a result of post‐MI‐induced stress hyperglycemia (18). Moreover, some of the hospitalized patients with hyperglycemia may have diabetes with poor blood glucose control, which will exacerbate the burden on the vital organs as well as increase mortality (19).

INR is an indicator of coagulation function (20). In the present study, patients with higher INR had a higher risk of severe AKI, which may be due to anticoagulant drugs used by patients with AMI. Excessive anticoagulation results in anticoagulation‐associated nephropathy with common pathological manifestations including hemorrhage and necrosis (21, 22).

The kidneys play an important role in maintaining electrolyte balance and regulating calcium ion metabolism (23). It is unclear whether blood calcium levels and calcium metabolism in the kidneys are associated, but one speculation is that early kidney damage affects calcium metabolism in the kidneys, which increases blood calcium levels. However, blood calcium levels at admission may be affected by several factors including prehospital fluid resuscitation and vasoactive drug use (24). Low blood calcium levels are an independent predictor of all‐cause mortality in critically ill patients with AKI (25); extremely high or low blood calcium levels may adversely affect patients with cardiogenic shock (26). Nevertheless, we did not find an association between high blood calcium levels and severe AKI.

Our study had several limitations. First, owing to several missing data in the database, complete data was not obtained and the patients with AMI were selected based on their discharge diagnoses records. However, data on factors, such as the AMI severity index and subsequent treatment, associated with the patients with AMI during admission, were not obtained, which may have affected the results. Second, AKI was diagnosed solely based on creatinine levels, which may have led to a loss of data on a population with AKI. Third, our sample was based on a retrospective analysis from a single center, which may be biased to a certain extent. Therefore, further multi‐center studies with a larger sample size should be conducted to obtain better results.

## 5 Conclusion

In patients with AMI, serious complications associated with AKI may increase in‐hospital mortality and the duration of stay in intensive care facilities. SBP; INR; and the levels of BUN, blood glucose, and calcium at the time of admission and a history of liver disease may be the independent risk factors for severe AKI in patients with AMI. Therefore, the identification of patients with AMI at risk of severe AKI should be improved through better monitoring and management of patients with AMI.

## Data availability statement

The datasets presented in this study can be found in online repositories. The names of the repository/repositories and accession number(s) can be found in the article/**Supplementary Material**.

## Ethics statement

The project was approved by the Institutional Review Boards of Beth Israel Deaconess Medical Center (Boston, MA) and the Massachusetts Institute of Technology (Cambridge, MA). The patients/participants provided their written informed consent to participate in this study.

## Author contributions

YN and DY contributed to the conception and design of the study. YN, XW, HQ, and JL organized the database. YN, JL, HY, and JC performed the statistical analysis. YN and XW wrote the first draft of the manuscript. DY, YF, HQ, HY, JC, and JY wrote sections of the manuscript. DY contributed leadership responsibility for the research activity planning and execution. All authors contributed to manuscript revision, and read and approved the submitted version.
