# Supplementary material for: Analysis of risk factors for severe acute kidney injury in patients with acute myocardial infarction: A retrospective study
Source: Front Nephrol. 2023 Feb 9;3:1047249. doi: 10.3389/fneph.2023.1047249 (PMC10479598; doi:10.3389/fneph.2023.1047249)
Supplement: Supplementary file 1 [file DataSheet_1.pdf]

Supplementary Material

Supplementary material Table 1. Diagnostic ICD code for patients with acute myocardial infarction

ICD 9 code	Classification of diagnosis
410.00-410.02	Acute myocardial infarction; of anterolateral wall
410.20-410.22	Acute myocardial infarction; of inferolateral wall
410.30-410.32	Acute myocardial infarction; of inferoposterior wall
410.40-410.42	Acute myocardial infarction; of other inferior wall
410.10-410.12	Acute myocardial infarction; of other anterior wall
410.50-410.52	Acute myocardial infarction; of other lateral wall
410.60-410.62	Acute myocardial infarction; true posterior wall infarction
410.70-410.72	Acute myocardial infarction; subendocardial infarction
410.80-410.82	Acute myocardial infarction; of other specified sites
410.90-410.92	Unspecified site

Supplementary Tables 2. Diagnostic code for the patient's complications

ICD 9 code	Classification of diagnosis
420-429	Congestive heart failure
430-438	Cerebrovascular disease
490-496	Chronic obstructive pulmonary disease
570 -571,0706,0709,5733,5734, 5738,5739, 0700-0709,4560,4562, 5722-5728	Liver disease
342-343,3341,3440-3446,3449	Hemiplegia / paraplegia
140-172,1740-1958,200-208,2386	Cancer

Supplementary Tables 3. Variable data missing and proportion

Variable	Number of missing	Proportion
Age	322	15.92%
Race	0	0.00%
Marital status	0	0.00%
Admission type	0	0.00%
Gender	0	0.00%
Hematocrit (%)	0	0.00%
Hemoglobin (g/dL)	0	0.00%
Anion gap (mmol/L)	7	0.35%
Bicarbonate (mmol/L)	4	0.20%
Blood Urea Nitrogen (mg/dl)	0	0.00%
Calcium (mg/dl)	247	12.22%
Chloride (mmol/L)	2	0.10%
Creatinine (mg/dl)	1	0.05%
Glucose (mg/dL)	16	0.79%
Sodium (mmol/L)	2	0.10%
Potassium (mmol/L)	1	0.05%
International normalized ratio (s)	97	4.80%
Heart rate (bmp)	6	0.30%
Systolic blood pressure (mmHg)	10	0.49%
Diastolic blood pressure (mmHg)	10	0.49%
Respirate rate (bmp)	7	0.35%
Temperature (℃)	123	6.08%
SPO_2 (mmHg)	11	0.54%
Congestive heart failure	0	0.00%
Cerebrovascular disease	0	0.00%
Chronic pulmonary disease	0	0.00%
Liver disease	0	0.00%
Hemiplegia or paraplegia	0	0.00%
Cancer	0	0.00%

Note: The missing values matched the random miss model and were filled by using "Predictive mean matching (PMM)" in the MICE package, as described previously[1].

[1] Z. Zhang: Multiple imputation with multivariate imputation by chained equation (MICE) package. *Ann Transl Med*, 4(2), 30 (2016) doi:10.3978/j.issn.2305-5839.2015.12.63
